# An Anterior Cingulate Cortex Neuronal Ensemble Controls Contextual Opioid Analgesic Tolerance

**DOI:** 10.1101/2025.08.16.670663

**Published:** 2025-08-19

**Authors:** Rafael E. Perez, Yann S. Mineur, Cheryl Chen, Wen-Liang Zhou, Julian L. Thompson, Saagar S. Motupally, Angélica Minier-Toribio, Marina R. Picciotto

**Affiliations:** Division of Molecular Psychiatry, Yale University School of Medicine, New Haven, CT, USA

## Abstract

Despite well-established cellular and molecular adaptations, opioid analgesic tolerance can be rapidly reversed in settings where these drugs are not expected. The specific neuronal populations that orchestrate this expectation-based tolerance remain poorly defined. In this study, we used a contextual tolerance training method alongside whole-brain clearing and immunostaining to identify brain regions involved in contextual tolerance and to pinpoint a specific neuronal ensemble in the ACC activated by this process. We observed that calcium activity in principal neurons of the ACC is suppressed by fentanyl in opioid-naïve mice or during contextual reversal but not during contextual tolerance. Chemogenetic silencing of the ACC induced tolerance reversal in the opioid-associated context without affecting thermal nociception in opioid-free mice. Furthermore, chemogenetic activation of the ACC contextual tolerance-active neuronal ensemble triggered analgesic tolerance in an unassociated context. This research highlights a role for ACC neuronal ensembles in mediating expectation-driven, contextual opioid analgesic tolerance without affecting basal nociception. Therefore, modulating the ACC could provide a promising strategy to improve pain relief while maintaining the essential ability to detect harmful stimuli.

## Results

**Figure 1.**
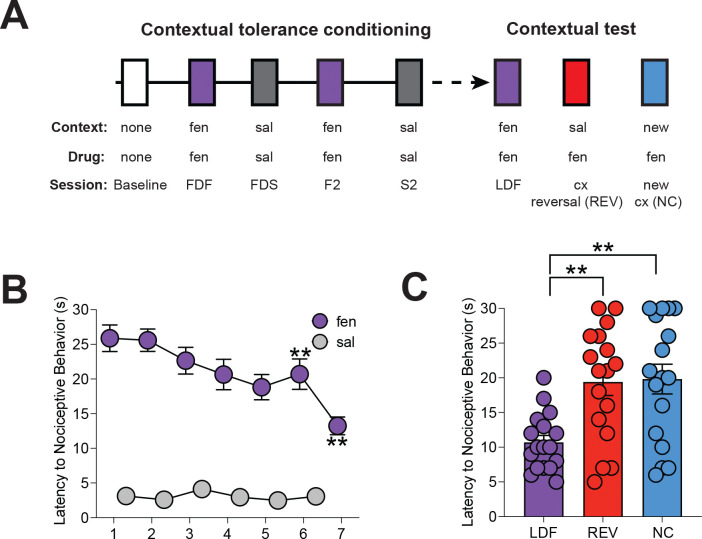
A conditioning procedure that induces contextual opioid analgesic tolerance in male mice. **A.** Experimental design: Mice received alternating daily injections of fentanyl (fen) or saline (sal) for 14 days in distinct contexts (cx), followed by daily assessment of antinociceptive response on a hotplate at 56°C. After conditioning, contextual tolerance was tested by administering fen in the previously sal-paired or a new cx and evaluating nociceptive latencies. **B.** A decrease in the antinociceptive effect of fen was observed over time, with reductions notable during sessions 5 and 7 when compared to the first fen session (FDF). No changes in nociception were observed after repeated sal injections. **C.** Mice showed a statistically significant reversal of analgesic tolerance compared to the Last Day of Fentanyl administration (LDF) in the sal-paired context (REV) or in a new cx (NC; n = 17).

Opioid analgesics rapidly lose efficacy due to the development of tolerance(1), which is more pronounced when the drug is administered repeatedly in the same context(2). Interestingly, tolerance can be reversed abruptly if opioids are administered in an unexpected context(2, 3). To investigate the mechanisms underlying this phenomenon, we established a novel behavioral model of contextual control of analgesic tolerance. Mice receive daily injections of fentanyl (25 μg/kg) in one context, alternating every other day with saline injections in a different context (Fig. 1A). Fentanyl initially increased latency to display nociceptive behaviors in the hot plate test. This analgesic response decreased by 50% after 7 fentanyl sessions, indicating the development of tolerance (Fig. 1B). We then tested whether tolerance was dependent on expectation of the drug by injecting mice with fentanyl and placing them in the saline-associated context or a new context. We found that placement in the saline-associated context or a new context before hotplate exposure significantly reversed tolerance (Fig. 1C). Thus, this paradigm reliably induces reversible context-dependent tolerance and is sensitive to the expectation of outcome before exposure to a nociceptive experience.

**Figure 2.**
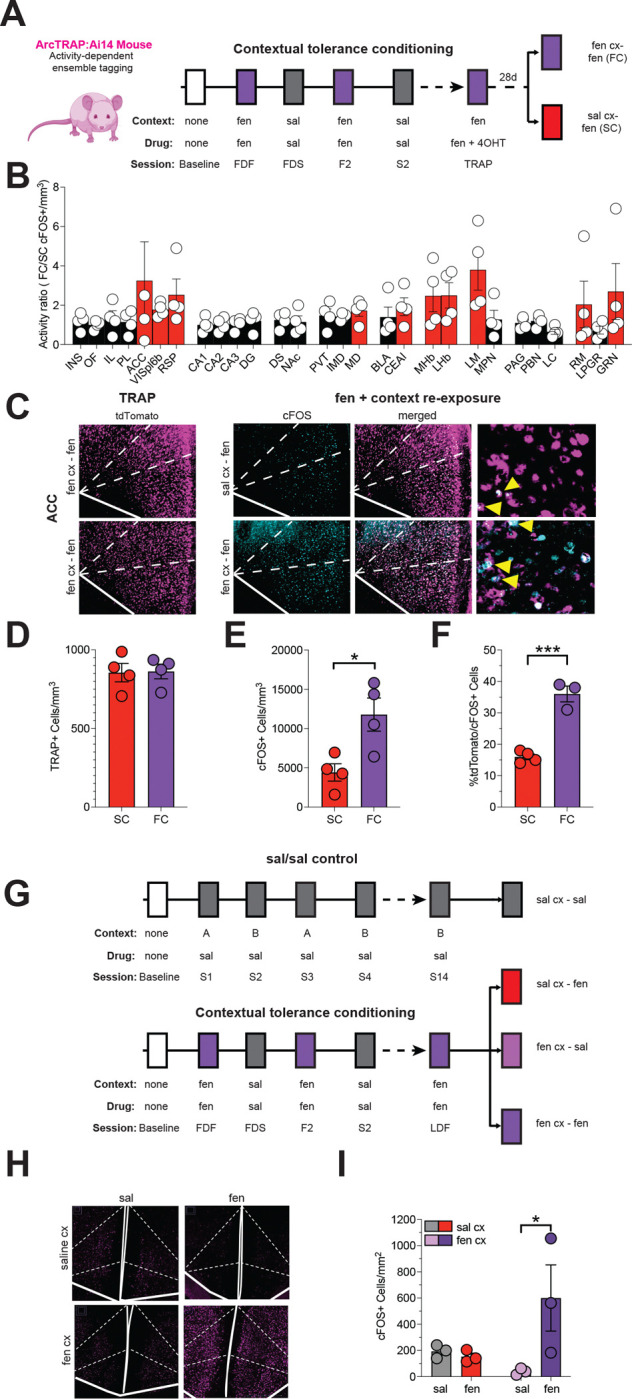
Contextual opioid tolerance induces brain-wide activity changes and the recruitment of an ACC neuronal ensemble. **A.** Experimental design for whole-brain activity mapping: ArcTRAP: Ai14 mice were administered 4-hydroxytamoxifen (4OHT, 50 mg/kg) on the last day of fen administration in the fen-paired cx (TRAP). Four weeks later, mice were re-exposed to fen in either the fen (FC) or sal cx (SC). **B.** Brain regions with increased ratio of cFos/tdTomato expression (FC / TRAP) following re-exposure to fen in the previously fen-paired cx. **C.** Representative images showing tdTomato+ (TRAP) and cFos+ cells in the ACC at 20x and 60x magnification. **D.** There was no difference in the number of TRAP cells across groups, consistent with equivalent fen and cx exposure in all mice during initial ensemble trapping. **E-F.** There was an increase in cFos+ and cFos+/TRAP cells in the ACC of mice re-exposed to fen in the previously fen-paired cx (n = 4 per group). **G**. Experimental design for evaluation of cFos labeling in response to cx, fen, or the combination of cx + fen. Control mice received sal in alternating cxs for 14 days (S1-S14), and experimental animals underwent fentanyl contextual tolerance conditioning before exposure to the previously fen-paired cx alone, fen alone, or the fen-paired cx + fen. **H.** Representative microscopy images showing cFos+ cells in ACC. **I.** There was a significant increase in cFos labeling in ACC following fen administration in the fen-paired cx **(**n = 3 per group; *p < .05, **p < .01, ****p < .0001). Data are shown as means ± SEM and individual data points. INS = insular cortex, OF = orbitofrontal cortex, IL = infralimbic cortex, PL = prelimbic cortex, VISpl6b = posterolateral visual cortex layer 6b, RSP = retrosplenial cortex, CA1 = hippocampal area CA1, CA2 = hippocampal are CA2, DG = hippocampal area dentate gyrus, DS = dorsal striatum, NAc = nucleus accumbens, PVT = paraventricular nucleus of the thalamus, IMD = interomediodorsal thalamus, MD = mediodorsal thalamus, BLA = basolateral amygdala, CEAl = lateral central nucleus of the amygdala, MHb = medial habenula, LHb = lateral habenula, LM = Lateral mammillary nucleus, MPN = medial preoptic nucleus, PAG = periaqueductal gray, PBN = parabrachial nucleus, LC = locus coeruleus, RM = nucleus raphe magnus, LPGR = lateral paragigantocellular nucleus, GRN = Gigantocellular reticular nucleus.

We then marked brain-wide ensembles of neurons active during contextual tolerance and its reversal in ArcCreER:Ai14 (ArcTRAP:tdTomato) mice (4), and used cFos labeling following whole brain clearing to identify cells active after re-exposure to either the fentanyl- (tolerance) or saline-paired (reversal) context (Fig. 2A)(5). Exposure to the fentanyl-associated context increased the number of activated neurons in brain areas known to be involved in memory, opioid hyperalgesia, and physiological homeostasis (Fig. 2B, sFig. 1B, Supplementary Table 1)(6–9). Interestingly, tolerance reversal increases neuronal activity in brain regions implicated in environmental novelty detection and contextual memory suppression, such as the perirhinal cortex (sFig. 1C, Supplementary Table 2)(10, 11). Beyond identifying brain regions involved in contextual tolerance, these findings suggest that reversing tolerance by changing context likely relies on an active process via the recruitment of brain regions involved in novelty detection(12).

We confirmed the whole-brain study findings using cFos labeling in candidate regions with 4 conditions controlling for independent effects of drug and context alone (Fig. 2G, sFig. 2). In both studies, the anterior cingulate cortex (ACC) was highly activated only when fentanyl was administered in the fentanyl-paired context (Fig. 2E, I). In addition, there was a 40% co-localization between ACC cells tagged during the last day of fentanyl training (tdTomato) and those labeled after re-exposure to fentanyl in the fentanyl context (cFos) four weeks later, suggesting that these cells could represent a persistent tolerance-sensing neuronal ensemble (Fig. 1D–F). This is of particular interest since the ACC has a central role in expectation-based pain modulation, such as contextual nocebo and placebo effects, as well as in opioid analgesia(13, 14).

**Figure 3.**
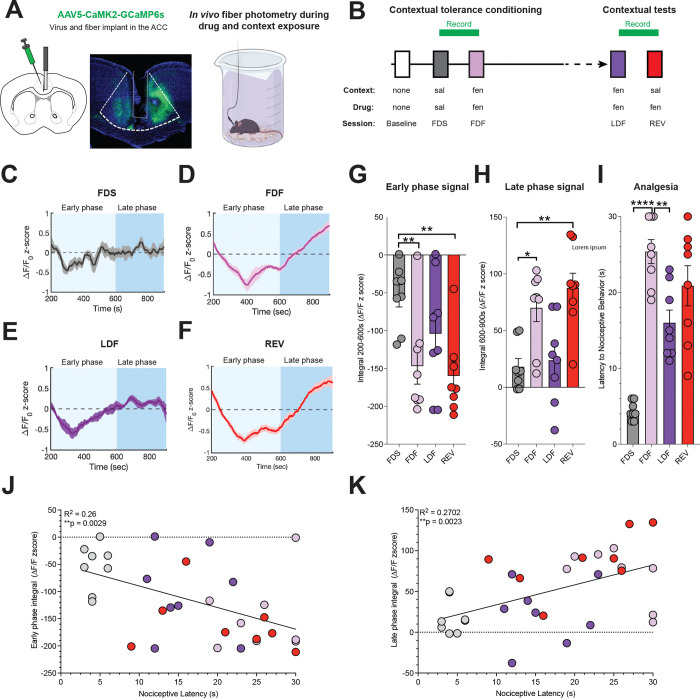
Correlation between ACC activity and fentanyl-induced antinociception across the development of contextual tolerance and during tolerance reversal. **A.** Schematic and representative image illustrating expression of the calcium indicator GCaMP6 and the implanted fiber in the anterior cingulate cortex (ACC). **B.** Timeline of experimental procedures. Each day, mice were administered either sal or fen, connected to the fiber patch cord, and placed in the sal- or fen-paired cx for 15 minutes before nociceptive testing on the hotplate. **C-F.** Photometric trace depicting ACC calcium signals. **C.** First Day of Saline (FDS) administration. **D.** First Day of Fentanyl (FDF) administration. **E.** Last Day of Fentanyl (LDF) conditioning and **F.** contextual reversal (REV, fen administered in the sal-paired cx). **G, H.** Quantification of early and late phase photometric responses. **G.** The magnitude of photometric signals during the early phase of context exposure was significantly reduced in both FDF and REV sessions compared to FDS and LDF. **H.** Conversely, the magnitude of calcium signals during the late phase was increased in FDF and REV sessions compared to FDS and LDF. **I.** Tolerance to the antinociceptive effects of fentanyl was evident during the LDF session, with a subsequent reversal observed during REV. **J.** A negative correlation was found between the calcium signal during the early part of the contextual exposures and nociceptive behaviors. **K.** A positive correlation was identified between the calcium signal during the late part of the contextual exposure and nociceptive behaviors, n = 8 (*p < .05, **p < 0.01, ***p < 0.001. Data displayed as means ± SEM and individual data points.

To evaluate the role of the ACC in the development and expression of contextual tolerance, we used *in vivo* fiber photometry to measure calcium transients with the calcium reporter GCamP6 (AAV5-CaMK2-GCaMP6f) as an indicator of neuronal activity in ACC neurons of behaving mice (Fig. 3A, B)(15). Fluorescent signals were measured during contextual tolerance conditioning and reversal testing (Fig. 3C–F). Compared to the first saline session (Fig. 3C), the first fentanyl dose during context exposure initially suppressed ACC activity (Early Phase), then caused a rebound in activity exceeding baseline levels several minutes later (Late Phase; Fig. 3D)(16). By the 7th fentanyl training session, the Early Phase suppression of ACC activity was lessened, and the Late Phase increase in activity was mostly absent (Fig. 3E). Both effects were restored when fentanyl was administered in the previously saline-paired context (Fig. 3F). These photometric patterns correlate with the levels of fentanyl-induced analgesia and tolerance (Fig. 3I), with a significant negative correlation observed between the magnitude of the Early Phase signal and the nociception response in the hotplate (Fig. 3G, J). Conversely, a positive correlation was found between the Late Phase signal and nociception (Fig. 3H, K).

In contrast to calcium activity changes in the fentanyl-paired context, we only observed decreases in ACC activity in the hotplate after the first fentanyl training session (sFig 3B). This effect was diminished following repeated exposure and was not restored by contextual reversal (sFig. 3C–D). These findings suggest that ACC activity during hotplate exposure is not involved in contextual analgesic tolerance reversal. Although we saw tolerance in fentanyl’s ability to suppress ACC signals across sessions on the hotplate, this was not dependent on context. Together, the findings from photometry experiments indicate that anticipatory ACC activity during **context exposure** is key for contextual analgesic tolerance and its reversal.

**Figure 4.**
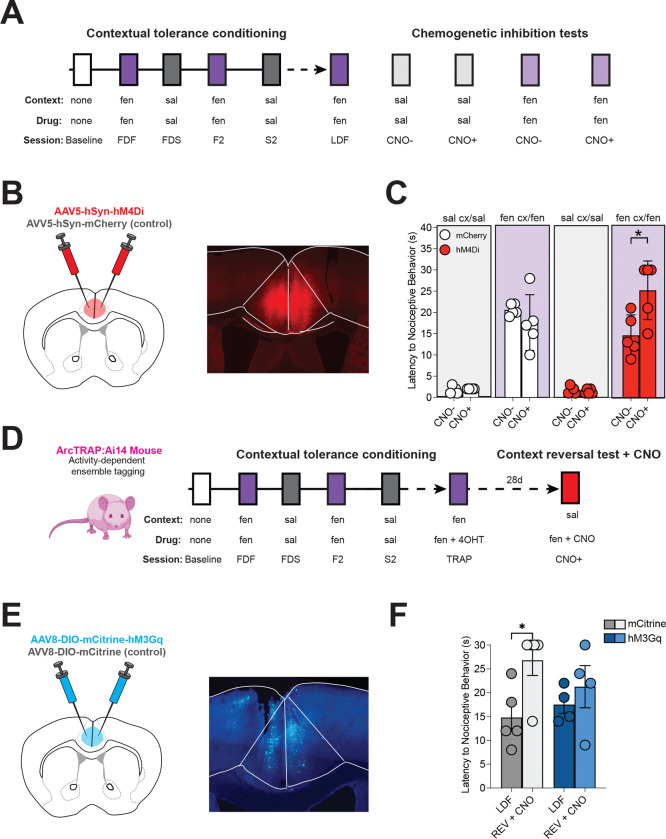
ACC tolerance-active ensemble neurons mediate contextual tolerance to fentanyl analgesia. **A.** Timeline and experimental design for the chemogenetic inhibition experiment. After mice underwent contextual analgesic tolerance training, they were exposed to either sal in the sal-paired cx or fen in the fen-paired cx, with or without the chemogenetic ligand clozapine-N-oxide (CNO). **B.** Schematic and representative image of viral injection of hsyn-hM4Di (hM4Di) in the ACC. **C.** Administration of CNO had no effect on nociceptive responses on the last day of sal administration in the sal cx in control or hM4Di-expressing mice, or fen administration in the fen cx in control mice, but resulted in increased nociceptive latencies in hM4Di-expressing mice following the last day of fen exposure in the fen cx, n = 5 per group. **D.** Experimental timeline. Mice underwent contextual analgesic tolerance conditioning until they developed tolerance, and on the last day of fen administration, were injected with the TRAP ligand 4OHT to induce CRE expression in active cells. Four weeks after 4-OHT administration, mice were placed in the sal-paired context and administered CNO and fentanyl (TRAP). **E.** Schematic of strategy to stimulate tolerance-active ensembles in the ACC. Male ArcTRAP mice were injected with viruses containing a cre-dependent chemogenetic activator, hM3Gq, or an mCitrine control. Representative image showing hM3Gq expression in the ACC. **G.** CNO administration had no effect in control mice but prevented contextual tolerance reversal in hM3Gq-expressing mice, n = 4 per group (*p < 0.05). Data displayed as means +/− SEM and individual data points.

To assess whether ACC activity and the context- and fentanyl-active ACC neuronal ensemble are necessary and sufficient for contextual analgesic tolerance, we employed a chemogenetic method to either inhibit all ACC neurons (Gi-DREADD, AAV5-hsyn-hM4Di-mcherry) or selectively activate the contextual tolerance neuronal ensemble (AAV2-DIO-hM3Gq-mCitrine in ArcTRAP mice) (Fig. 4A, B). Administration of the DREADD ligand clozapine-N-oxide (CNO) did not alter nociception in response to saline administration or when fentanyl was administered to control mice. However, CNO reversed tolerance in Gi-DREADD-expressing mice when fentanyl was administered in the fentanyl-paired context (Fig. 4C). Thus, inhibiting ACC activity blocks contextual tolerance without altering nociception. We next tested whether reactivating ACC tolerance-active ensemble neurons is sufficient to induce tolerance in an unassociated context (Fig. 4D, E). Ensemble neurons were tagged on the last day of fentanyl exposure as described above, with one notable exception: mice were not placed on the hot plate after fentanyl and contextual exposure to avoid labeling ACC cells that encode nociception(4, 17). Three weeks after ensemble labeling, mice were administered fentanyl and CNO in the previously saline-paired context (Fig. 4F). As expected, control mice showed reversal of analgesic tolerance after exposure to the saline-paired context. However, excitation of ACC-tagged ensembles in Gq-DREADD-expressing mice with CNO was sufficient to maintain fentanyl tolerance (prevent reversal) in the saline-paired environment (Fig. 4F). Therefore, selective modulation of ACC neuronal populations can either induce or prevent contextual analgesic tolerance without affecting nociception.

In this study, using a novel conditioning procedure in mice, we show that ACC activity encodes anticipation of drug analgesic responses in an associated context to induce tolerance that can be quickly reversed despite profound molecular and cellular adaptations(1). We used this paradigm to show that the activity of principal ACC neurons tracks the development and expression of contextual opioid tolerance. Consistent with these changes in ACC, chemogenetic regulation of ACC neuronal ensemble activity shows that it is sufficient for contextual tolerance and necessary for its reversal. ACC regulation of contextual analgesic tolerance is specific to the administration of opioids in the opioid-paired context, a condition in which the drug is expected. These results align with previous work showing the ACC plays a crucial role in regulating anticipated pain and analgesia(13, 18, 19). In contrast to a recent study that compared morphine analgesic tolerance in mice receiving repeated escalating doses of morphine either in their home cage or in a different context (Hou *et al*)(20), the current experiments identify the ACC as a critical mediator of tolerance in a paradigm that measures expectancy as an important parameter in both contextual tolerance and its reversal(20).

The current findings align with previous research showing that ACC is necessary for learned expectations of pain, pain avoidance, and pain relief(18, 19, 21, 22). These results build on earlier work by being the first to identify the role of the ACC in expectation-based analgesic tolerance, independent of pain modulation. We found that ACC activity increases only when both context and drug are presented together, and not after saline treatment, the condition with the highest degree of nociception. Further, chemogenetic silencing of ACC activity did not change nociceptive responses in mice treated with saline. Additionally, stimulating an ACC fentanyl-active neuronal ensemble tagged in the absence of a pain experience (no hotplate exposure) is sufficient to prevent reversal of analgesic tolerance in a saline-paired environment. Together, these findings indicate that ACC modulation of tolerance extends beyond alteration of primary nociception, consistent with reports from chronic pain patients who have undergone cingulotomies, who state that they do not notice changes in sensation, but instead note a decrease in pain unpleasantness(23). ACC activity is also essential for conditioned placebo and nocebo responses without noxious stimuli(13, 18) , as well as socially observed pain and analgesia(24). These conditioned and empathic responses reflect the expectation of future pain and analgesia, consistent with the expectation of analgesia that occurs in the current conditioning paradigm.

In summary, we report a novel role for an ACC neuronal ensemble in mediating contextual opioid analgesic tolerance without affecting basal nociception. Modulating ACC activity presents a promising opportunity for rapidly reversing analgesic tolerance in environments associated with drug use, positioning targeted interventions in this region as a potential therapeutic strategy to enhance pain relief while preserving the essential ability to detect and respond to harmful stimuli.

## Materials and Methods

### Animals

All animal experiments were approved by the Yale University Institutional Animal Care and Use Committee. Male C57BL/6J mice were purchased from Jackson Laboratories. Arc- and tamoxifen-dependent Cre transgenic (ArcTRAP) mice (B6.129(Cg)-*Arc*^*tm1.1(cre/ERT2)Luo*^/J) were used for ensemble manipulation experiments. ArcTRAP mice were crossed in-house with tdTomato (B6.Cg-*Gt(ROSA)26Sor*^*tm14(CAG-tdTomato)Hze*^/J) or eYFP (B6.129X1-*Gt(ROSA)26Sor*^*tm1(EYFP)Cos*^/J) reporter mice to label active populations in whole-brain imaging and immunohistochemistry experiments. Mice were maintained on a 12-h light/dark cycle (lights on 7:00) with access to food and water *ad libitum*. Experiments were conducted from 10:00–18:00.

### Stereotaxic surgeries and injections

Mice (~8–12 weeks old) were anesthetized with isoflurane (4% for induction, 1.5–1% for maintenance) in a stereotaxic frame (Kopf). Adeno-associated viral (AAVs) vectors were injected into the ACC (anteroposterior (AP) +1.00 mm; mediolateral (ML) +/− 0.35 mm; dorsoventral (DV) −1.80 mm). AAVs were injected using a manual 10 ml Hamilton microsyringe. The needle was lowered to the target, and the virus was injected at 100 nL/min. The needle remained in place for 10 min before being withdrawn. For fiber photometry studies, AAVs containing the calcium sensor GCaMP6f (AAV5-CaMKII-gCaMP6f) were injected into the ACC unilaterally in a random order (400 nL). Optical fiber implants consisted of a zirconia ferrule (Doric Lenses) fitted with an optical fiber (200 μm core diameter, NA = 0.37), lowered to 0.1 mm above the injection DV immediately after viral injection. The ferrule was fixed to the skull using dental cement (3M). For the chemogenetic inhibition experiment, AAVs containing the fluorescent reporter mCherry (AAV5-hSyn-mCherry) or hM4Di (AAV5-hSyn-DIO-hM4D(Gi)-mCherry) were injected bilaterally (800 nL). AAV constructs were purchased from Addgene. Mice were given carprofen analgesic (5 mg/kg, intraperitoneal) and allowed to recover for at least 3 weeks before behavioral experiments. For ensemble stimulation experiments, AAV constructs containing a cre-dependent hM3Gq (AVV8-hSyn-DIO-HA-hM3D(Gq)-IRES-mCitrine) from Addgene or mCitrine (AV2/8-hSYN-DIO-HA-IRES-mCitrine) were injected into the ACC of ArcTRAP mice. Implant sites and viral expression were verified after behavioral experiments using histology. Mice that had poor or mistargeted viral expression were excluded from data analyses (n = 4). Viral expression and implant targeting in mice used in experiments is shown in Supplementary Figure 4.

### Conditioned opioid analgesic tolerance procedure and hotplate test of thermal nociception

Before all behavioral experiments, mice were handled and habituated to subcutaneous injections. Mice then underwent tolerance training for 14 days. On each behavioral training day, mice were acclimated to the testing room for 1 hour before testing. Fentanyl or saline injections were randomly associated with one of two contexts with distinct multisensory features. One context consisted of a clear beaker with a flat black metal floor, was infused with an orange scent and kept in a brightly lit room. The other context consisted of a beaker lined with black and white vertical stripes and a soft gray mesh wire floor, was infused with almond scent and kept in a dimly lit room. During training, mice received daily alternating subcutaneous injections of either fentanyl (25 μg/kg, NIDA Drug Supply Program) or saline before being placed in the respective context for 15 minutes. One day after training, antinociception and analgesic tolerance were tested by injecting mice with fentanyl and placing them in the fentanyl-associated context. Contextual tolerance reversal was tested by injecting mice with fentanyl and placing them in the saline-associated context.

Following context presentations, mice were placed on the hotplate at 56.0 +/− 0.5°C (Columbus Instruments). Mice were removed from the hotplate when nocifensive behaviors such as hind paw licking, withdrawing, flailing, and jumping were observed or after 30s (to avoid tissue damage). Mice that showed paw injury or any other type of injury before any training or testing session were excluded from data analyses (n = 6). Hot plate sessions were recorded throughout conditioning and during contextual reversal tests. Saline and fentanyl conditioning sessions were scored unblinded to ensure only mice that achieved tolerance were used in contextual tolerance tests. Contextual test sessions were scored by an investigator blinded to context, drug, genotype, or virus condition.

For context and drug exposure experiments, 24 h after the last tolerance training session, mice were exposed to one of four conditions for 15 mins before hotplate testing: saline in the saline-associated context, saline in the fentanyl-associated context, fentanyl in the saline-associated context, and fentanyl in the fentanyl-associated context. 90 min after context pairings, mice were transcardially perfused, and brains were extracted and processed for immunohistochemistry.

### Whole-brain activity-dependent labeling and mapping

Male arcTRAP:Ai14 mice were injected with 4-OHT (Sigma-Aldrich) at 50 mg/kg (dissolved in a 1:9 EtOH/peanut oil solution) 1 hr after the last tolerance training session to label tolerance-active cells. 4 weeks after ensemble labeling, mice were injected with fentanyl and re-exposed to either the previously saline- or previously fentanyl-paired context. 90 mins after the context presentation and hotplate test, mice were transcardially-perfused with ice-cold PBS containing 10 U/ml heparin, followed by 4% PFA. Brains were extracted and fixed in 4% PFA for 24 hours at 4 °C. Brains were cleared and processed by LifeCanvas Technologies using the SHIELD (stabilization under harsh conditions via intramolecular epoxide linkages to prevent degradation) protocol. Briefly, tissues were cleared for 7 days using Clear+ delipidation buffer and labeled using SmartBatch+ with rabbit anti-FOS (Abcam). Fluorescently conjugated secondary antibodies (Jackson ImmunoResearch) were applied in a 1:2 primary secondary molar ratios. Tissues were then incubated in EasyIndex (LifeCanvas Technologies) for refractive index matching (n = 1.52) and imaged using SmartSPIM. Images were registered to the Allen Brain Institute Autofluorescence Atlas and cells were quantified automatically using a neural network (LifeCanvas Technologies) as previously described.

### Immunohistochemistry

Mice were anesthetized with Fatal-Plus^®^ (Patterson Veterinary) and were transcardially perfused with ice-cold PBS, followed by 4% paraformaldehyde (PFA). Brains were extracted and post-fixed overnight in 4% PFA at 4 °C. 24 hrs after, brains were transferred to a 30% sucrose solution in PBS for up to 48 hours at at 4 °C. Brain were embedded in Tissue-Tek^®^ O.C.T. Compound (Sakura Finetek USA). Coronal brains sections were sliced on a sliding microtome (Leica) into at 40 μm thickness and collected into 12-well plates in PBS with 0.3% sodium azide for storage. For immunohistochemical processing, tissue was washed in PBS for 10 minutes 4 times. The tissue was then incubated with a blocking buffer consisting of 0.3% TritonX-100 in PBS (PBST) for 1 h and in PBST with 3% normal donkey serum for 1 h at room temperature. The brain sections were incubated with primary antibodies at 4 °C in a blocking buffer consisting of 1% normal donkey serum in 0.1% PBST for 24 h at room temperature. Slices were washed 4 times with PBS for 10 mins per wash and were incubated with secondary antibodies for 24 h at room temperature. After secondary antibody incubation, slices were washed with PBS 4 times and incubated with DAPI (Sigma) in PBS for 5 mins before being rinsed with PBS 4 times and being mounted onto super-frost Plus glass slides (VWR). Slices were imaged using a Fluoview FV10i microscope (Olympus) with 20x objective and 0.6 numerical aperture. Primary antibodies used for histology were: Rabbit anti-c-Fos (diluted 1:1000, Cell Signaling Technologies) and Chicken anti-GFP (diluted 1:1000, Abcam). Secondary antibodies used were Donkey anti-rabbit Alexa-Fluor 647 (diluted 1:1000, Thermo Fisher Scientific) and Donkey anti-Chicken Alexa-Fluor 555 (diluted 1:1000, Fisher Scientific).

### Fiber photometry

Mice were acclimated to the fiber photometry cable, and baseline- and foot shock-elicited calcium transients were recorded before contextual tolerance experiments to ensure signal quality. Mice that did not show foot shock-elicited elevations in photometric signals were excluded from further testing (n = 8). For photometry testing during contextual tolerance conditioning, mice were placed in a holding cage for 5 min. Mice were then injected with fentanyl or saline and placed in the appropriate context for an additional 15 minutes, during which signals were recorded. After exposure to the context, mice were immediately placed on the hot plate. Mice were removed from the hot plate 5 s after displaying nocifensive behaviors or after 35 s. Fluorescent signals were acquired and analyzed using custom-written MATLAB code. Signals were recorded using two LED lights at 405 and 465 nm (30 μW, Doric Lenses). The 405 channel was used as an isosbestic control, and the 465 channel was used as the calcium fluorescence channel. The first 100 s of recording were cropped to eliminate plugin artifacts from analysis(16). Change in fluorescence (ΔF/F) was calculated as (465 nm signal – fitted 405 nm signal)/465 signal at each time point. The Z-score for each session was calculated using the formula: Z = (x−y)/standard deviation. (where x = ΔF/F and y = mean of ΔF/F for baseline). Graphs were generated using the smooth data function on MATLAB with a running average of 6000. For context exposure analyses, the absolute signed integral for the first 600s (Early Phase) and the last 300s (Late Phase) of each 900s-context exposure was calculated using the trapezoidal method. For hot plate signal analyses, a 6-second window centered on hot plate placement was used, with the period from 2 seconds before placement to the moment of placement serving as the pre-placement baseline, and the subsequent period as the post-placement period.

### Chemogenetic regulation of conditioned opioid analgesic tolerance

For chemogenetic inhibition studies, male C57BL/6J mice expressing either mCherry control (AAV5-hSyn-mCherry) or Gi-DREADD virus (AAV5-hSyn-hM4Di-mCherry) in the ACC underwent contextual tolerance conditioning. Mice that showed tolerance on the last day of fentanyl training were used for chemogenetic testing. 24 hrs after the last conditioning session, chemogenetic testing was initiated. On the first day of testing mice were injected with saline intraperitoneally followed 30 mins later with a subcutaneous saline injection before being placed in the saline context (saline controls). In the next session mice were intraperitoneally injected with clozapine-N-oxide (CNO; 5 mg/kg, HelloBio) followed 30 minutes later with a subcutaneous saline injection immediately before being placed in the saline context. On the third testing session, mice received an intraperitoneal injection of saline, followed 30 mins later by fentanyl administration and placement in the fentanyl context. On the last testing session, mice were injected with CNO, and 30 mins later injected with fentanyl before being placed in the fentanyl context. For tolerance-active ensemble excitation studies, male ArcTRAP mice were stereotaxically injected with either cre-dependent mCitrine (AAV2/8-HA-DIO-mCitrine; control) or Gq-DREADD-mCitrine-containing (AAV2/8-HA-DIO-hM3Gq-mCitrine) viruses into the ACC. After 4 weeks of recovery, mice underwent contextual tolerance training. Mice that showed tolerance on the last fentanyl training session were used for further testing. 24 hr after the last training session, mice were injected with fentanyl and placed in the fentanyl context for 15 minutes before being returned to their home cage. 1 hr after context exposure, mice were lightly anesthetized and injected with the TRAP ligand 4-OHT (50 mg/kg)(4). 3 weeks after 4-OHT injection, mice were injected intraperitoneally with CNO, 30 mins later, mice were injected with fentanyl and placed in the previously saline-paired context for 15 minutes.

### Statistical analyses

Sample sizes for analyses are based on previously published studies that used similar approaches(4, 13, 18, 20). Data were analyzed using Prism 10 (GraphPad). All data are reported as mean ± SEM, with individual values superimposed on graphs. Statistical significance was tested using t-tests, one-way or two-way ANOVAs, and Sidak’s multiple comparisons *post hoc* test when main effects were detected. Repeated Measures were conducted for within-subject data when appropriate. P < 0.05 was considered statistically significant, with trends reported as exact p-values in the text.

## Supplementary Material

Supplement 1

Supplement 2

## Significance Statement

Opioids are first-line treatments for pain, but tolerance leads to decreased efficacy and escalated dosing, increasing opioid use disorder and overdose risk. Remarkably, after repeated administration, tolerance can be rapidly reversed when the drug is administered in a context where it is not expected. However, the neuronal mechanisms underlying tolerance based on contextual expectation are still not well understood. Here, we demonstrate that an anterior cingulate cortex neuronal ensemble is necessary and sufficient to orchestrate expectation-driven tolerance, suggesting that targeted inhibition of this ensemble could prevent tolerance and improve analgesic treatment.
