# Supplementary material for: An Anterior Cingulate Cortex Neuronal Ensemble Controls Contextual Opioid Analgesic Tolerance
Source: bioRxiv. 2025 Aug 19:2025.08.16.670663. Preprint. [Version 1] doi: 10.1101/2025.08.16.670663 (PMC12393370; doi:10.1101/2025.08.16.670663)
Supplement: Supplement 1 [file media-1.pdf]

Supplemental Figures

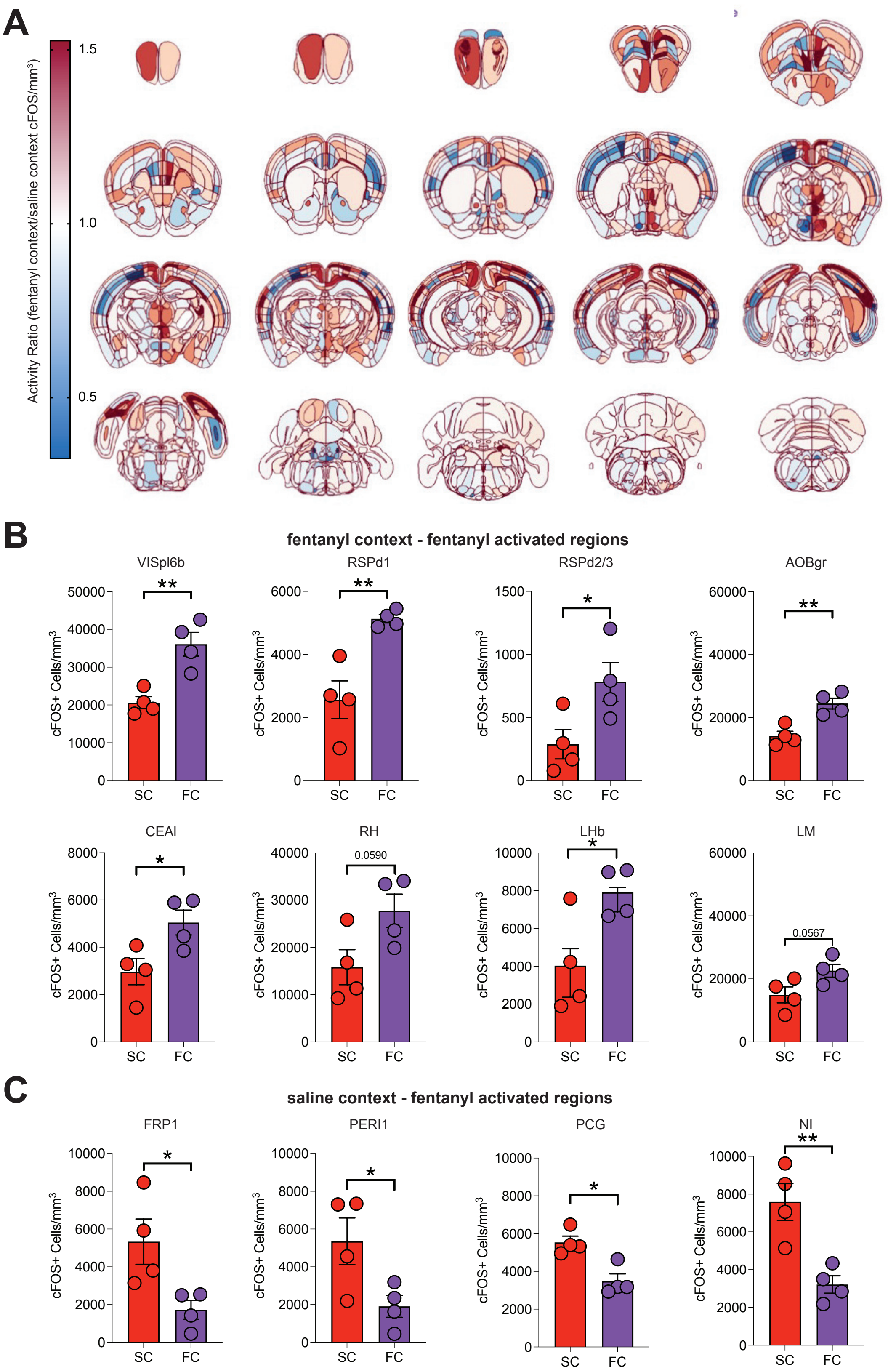

Supplementary Figure 1. Brain regions with significantly increased cFos activity after context re-exposure. A. Schematic showing differential cFos density across the brain, with red representing greater cFos

expression following fen exposure in the fen-paired cx (FC) and blue representing greater cFos expression following fen exposure in the sal-paired cx (SC). **B.** Brain areas with increased cFos labeling following fen and fen-paired cx re-exposure. **C.** Areas with increased cFos labeling following fentanyl and sal-paired cx re-exposure (n = 4 per group; * $p < 0.05$, ** $p < 0.01$). Data are shown as means ± SEM, individual data points. VISpl6b = Posterolateral visual area, layer 6b, RSPd1 = Retrosplenial area, dorsal part, layer 1, RSPd2/3 = Retrosplenial area, dorsal part, layer 2/3, AOBgr = Accessory olfactory bulb, granular layer, CEAl = Central amygdala nucleus, lateral part, RH = Rhomboid nucleus, LH = lateral habenula, LM = Lateral mammillary nucleus. FRP1 = Frontal pole, layer 1, PERI1 = perirhinal area, layer 1, PCG = Pontine central gray, NI = Nucleus incertus.

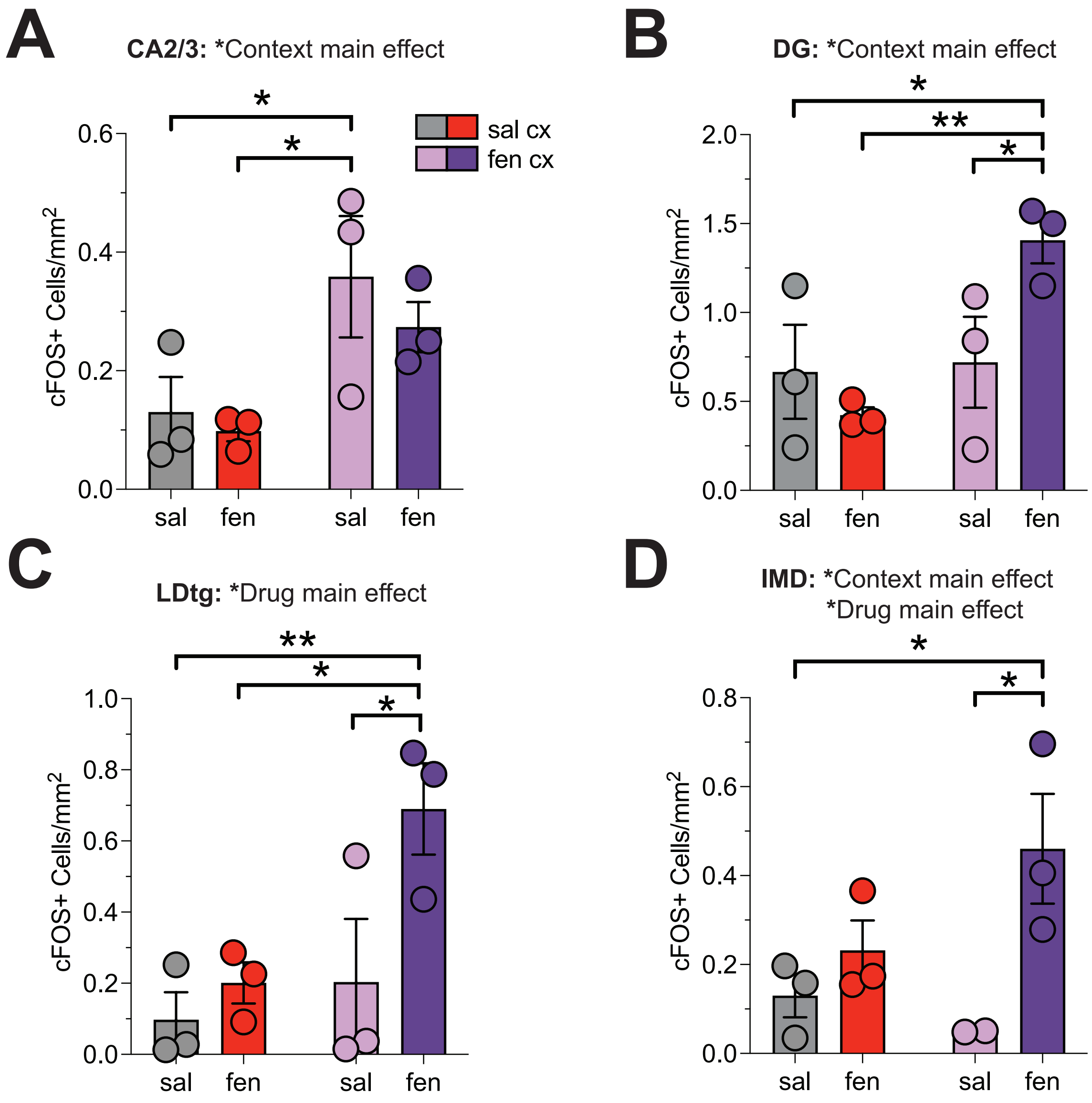

Supplementary Figure 2. Effects of context, drug, or context and drug on cFos expression. A. Fen-paired cx exposure in the absence of drug increased cFos labeling in the CA2/3 region of the hippocampus. **B.** cFos expression in the dentate gyrus (DG) subregion is significantly increased in response to exposure to the fen-paired cx and further increased in the cx + fen condition. **C.** There is a main effect of drug (fen exposure) on cFos labeling in the laterodorsal tegmentum (LDtg). **D.** Drug and cx have a main effect on cFos labeling in the interomediodorsal thalamus (IMD), n = 3 per group, (*p < .05, **p < .01). Data are shown as means ± SEM and individual data points.

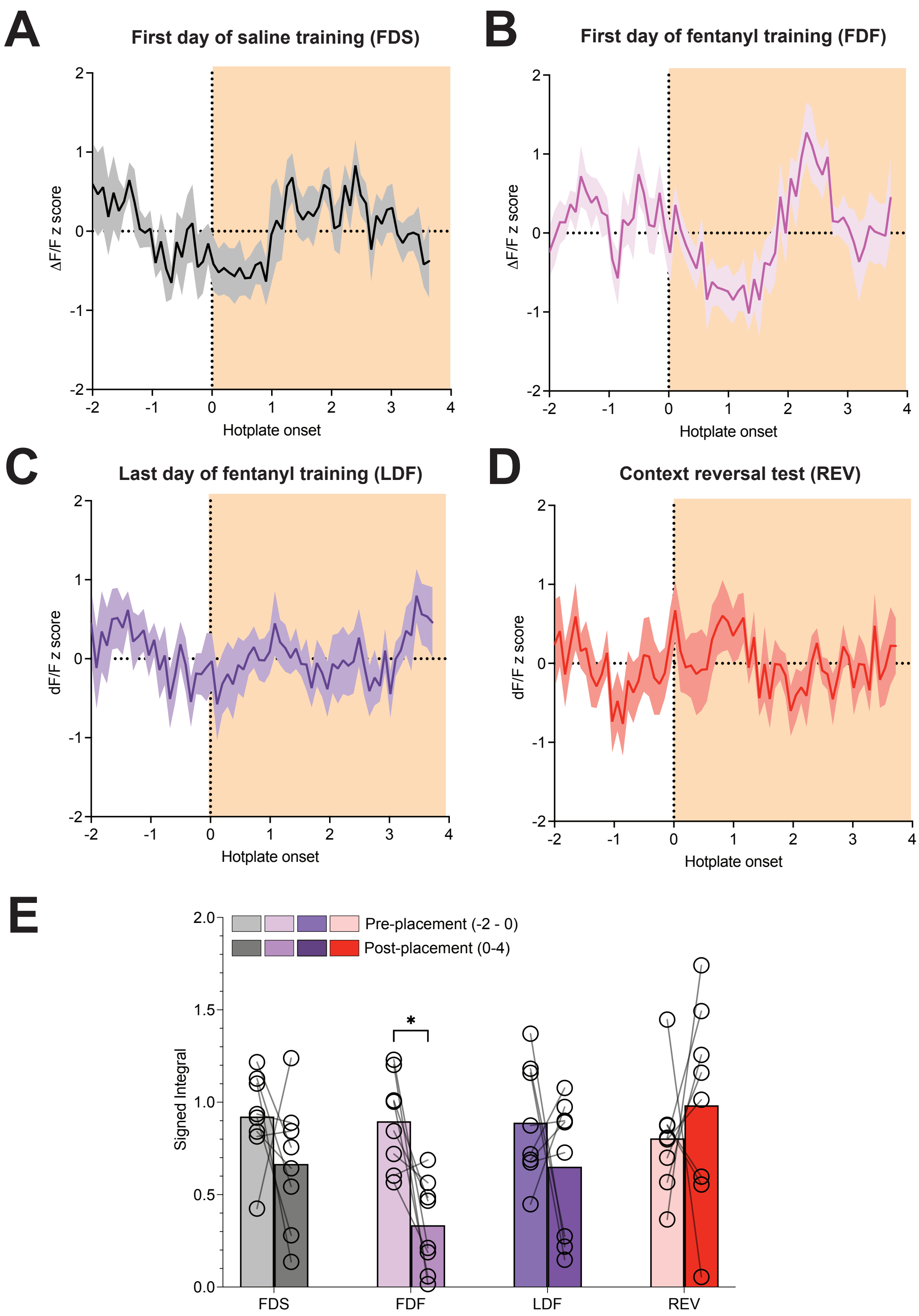

Supplementary Figure 3. ACC activity during the hotplate test immediately after contextual conditioning and tolerance reversal. A. Photometric trace depicting calcium signals during hotplate exposure on the First Saline Day (FDS). **B**. ACC calcium signals during hotplate exposure on the First Day of Fentanyl (FDF), and **C.** Last Day of Fentanyl (LDF) sessions. **D.** ACC calcium signals during the contextual reversal (REV) session. **E.**

There was a significant decrease in the signed integral of the photometric signal while mice were on the hotplate on the FDF, n = 8 (*p < .05). Data displayed as means ± SEM and individual data points.

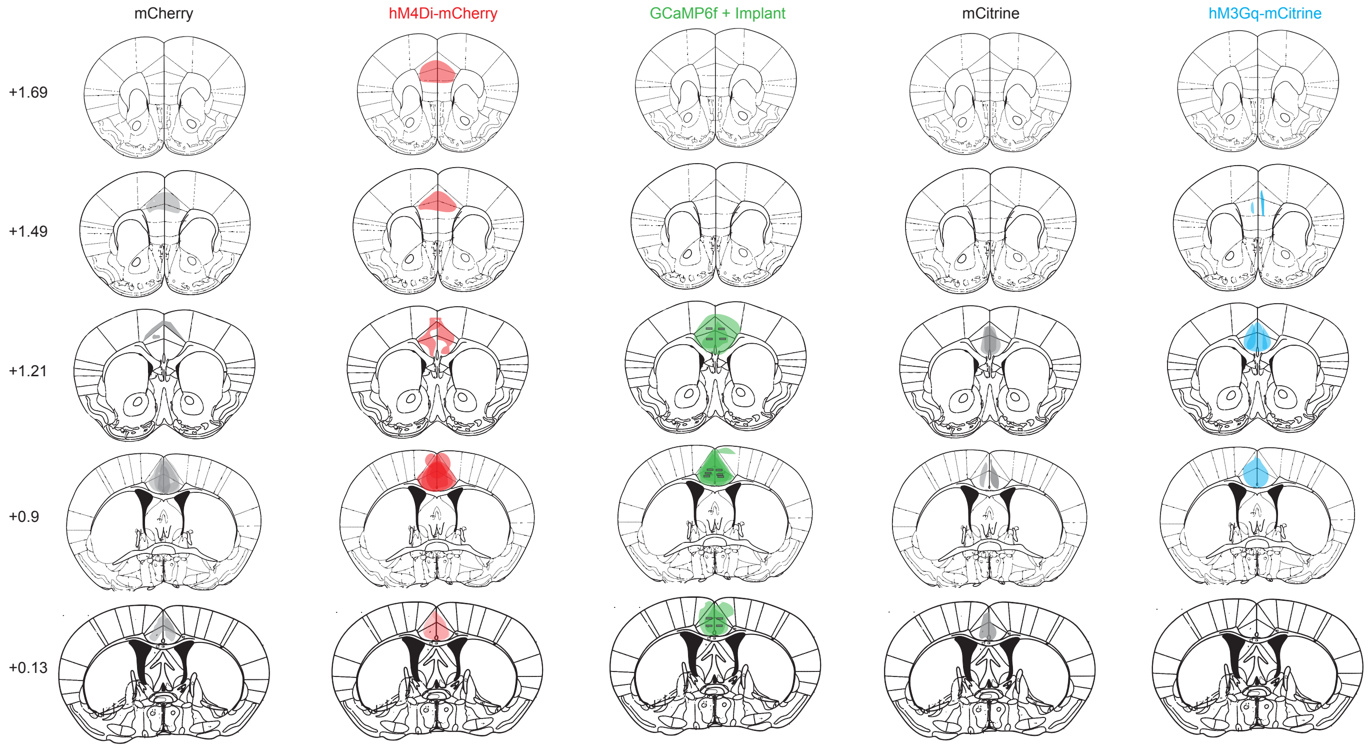

Supplementary Figure 4. Viral expression and implant placement for all experiments. A-B. On-target viral expression for mCherry and hM4Di in mice used in Figure 3C (2 mCherry- and 4 hM4Di mice were removed from analysis due to mistargeting or lack of viral expression). **C.** On-target GCaMP6f viral expression and bilateral implant location (hash marks) in mice used in Figure 2 (4 mice were removed from analysis due to a lack of viral expression). **D-E.** On-target viral expression for mCitrine and hM3Gq in mice used in Figure 3F (4 mCitrine- and 3 hM3Gq-infused mice were removed from analysis due to lack of viral expression).
